# Molecular contrast on phase-contrast microscope

**DOI:** 10.1038/s41598-019-46383-6

**Published:** 2019-07-18

**Authors:** Keiichiro Toda, Miu Tamamitsu, Yu Nagashima, Ryoichi Horisaki, Takuro Ideguchi

**Affiliations:** 1 Department of Physics, The University of Tokyo, Tokyo, 113-0033 Japan; 2 Department of Neurology, The University of Tokyo, Tokyo, 113-0033 Japan; 3 Graduate School of Information Science and Technology, Osaka University, Osaka, 565-0871 Japan; 4 PRESTO, Japan Science and Technology Agency, Saitama, 332-0012 Japan; 5 Institute for Photon Science and Technology, The University of Tokyo, Tokyo, 113-0033 Japan

**Keywords:** Infrared spectroscopy, Phase-contrast microscopy

## Abstract

An optical microscope enables image-based findings and diagnosis on microscopic targets, which is indispensable in many scientific, industrial and medical settings. A standard benchtop microscope platform, equipped with e.g., bright-field and phase-contrast modes, is of importance and convenience for various users because the wide-field and label-free properties allow for morphological imaging without the need for specific sample preparation. However, these microscopes never have capability of acquiring molecular contrast in a label-free manner. Here, we develop a simple add-on optical unit, comprising of an amplitude-modulated mid-infrared semiconductor laser, that is attached to a standard microscope platform to deliver the additional molecular contrast of the specimen on top of its conventional microscopic image, based on the principle of photothermal effect. We attach this unit, termed molecular-contrast unit, to a standard phase-contrast microscope, and demonstrate high-speed label-free molecular-contrast phase-contrast imaging of silica-polystyrene microbeads mixture and molecular-vibrational spectroscopic imaging of HeLa cells. Our simple molecular-contrast unit can empower existing standard microscopes and deliver a convenient accessibility to the molecular world.

## Introduction

An optical microscope is a universal tool permeated through numerous aspects of science, industry and medicine. Observation of microscopic world is at the basis of studying unknown physical^1–3^ and biological^4–6^ phenomena, mass-production inspection such as semiconductor^7^ and pharmaceutical^8^ production lines, and clinical and medical diagnosis^9–11^, in addition to daily research activities. Today, various microscopic modalities are offered by commercial benchtop platforms and add-on units including bright-field (BF), dark-field (DF), differential-interference-contrast (DIC), and phase-contrast (PC)^12^ modalities. These are powerful tools to obtain image contrast based on optical absorption, scattering or thickness revealing the microscopic morphology of the specimen, and yet convenient because the specimen can be observed, without any specific preparation or alteration, in real time or even at a high frame rate if equipped with a commercial high-speed camera.

Despite the above-mentioned superior capability on morphological imaging, standard BF microscopes are inherently blind to molecules and none of the existing add-on units, such as DF, DIC or PC, is able to provide molecular contrast (MC). Although vibrational spectroscopic techniques such as infrared absorption and Raman scattering spectroscopy can be considered as candidates for realizing a MC unit, it turns out that they are not compatible with a standard microscope, which is operated with wide-field incoherent visible light illumination and image acquisition with a camera. This is because an infrared absorption microscope requires a light source in the infrared spectral region, while Raman microscope a visible laser and a spectrometer. Furthermore, both microscope techniques are often operated in a point-scanning detection instead of wide-field imaging with an image sensor.

Here, we propose and demonstrate a concept of add-on MC unit to a standard benchtop optical microscope system, which is enabled by mid-infrared (MIR) photothermal effect induced by an amplitude-modulated mid-infrared semiconductor laser. The amplitude-modulated MIR laser beam illuminated widely onto the specimen generates the refractive-index modulation via photothermal effect^13–15^ at specific sites where vibrationally resonant molecules exist, which is then visualized by phase-sensitive imaging with a PC microscope. In this proof-of-concept demonstration, we develop a system, which we call MC-PC microscope, based on a commercial PC microscope platform and perform high-speed imaging of silica-polystyrene microbeads mixture at 3,000 frames per second (fps) and molecular-vibrational spectroscopic imaging of HeLa cells at 10 fps.

## Results

### Principle of MC-PC microscopy

**Figure 1:**
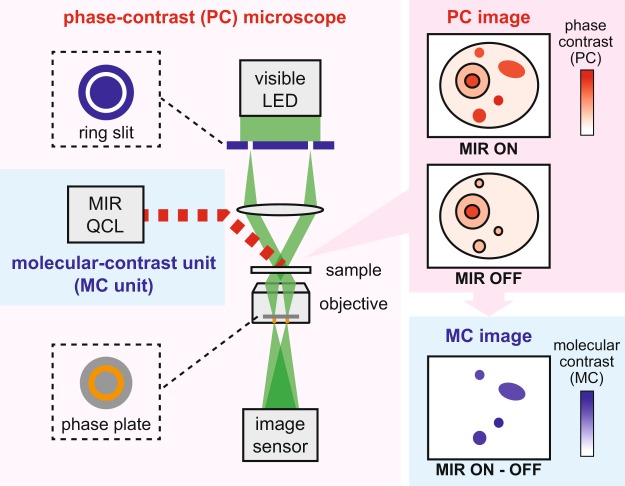
Molecular-contrast phase-contrast (MC-PC) microscopy. To obtain the MC image of the sample, the add-on “MC unit” is attached to an existing standard PC microscope platform, which consists of an amplitude-modulated continuous-wave MIR QCL. The amplitude-modulated MIR beam is weakly focused onto the sample placed at the objective focus to cover the entire region of the specimen. This induces the molecular-vibrational photothermal effect at specific sites within the specimen where vibrationally resonant molecules exist, which is detected as temporal intensity-modulations in the time-series of the recorded PC images. These photothermal signatures are computationally extracted to reveal and add the resonant MC to conventional PC microscope images. LED: light-emitting diode.

Our MC-PC microscope is realized by the MC unit, comprising of an amplitude-modulated MIR semiconductor quantum cascade laser (QCL)^16^, attached to a standard PC microscope platform (see Fig. 1). The amplitude-modulated MIR laser beam excites the wide-field area of the sample at the objective focus. The molecular-vibrational photothermal effect takes place only at specific sites in the sample where vibrationally resonant molecules exist, which appears as temporal intensity modulations in the PC image. The camera records the video of this phenomenon, and the resulting periodic photothermal signals are computationally extracted to produce the MC image. Note that a PC microscope, unlike, e.g., a BF microscope, is the key instrument to achieve the sensitive detection of the MC, because the photothermal refractive-index change appears in the optical phase rather than in the amplitude.

It is noteworthy that our MC-PC microscope can potentially realize high speed MC imaging with high spatial resolution and low photodamage. Although the MC is resulting from MIR absorption, the spatial resolution of our MC images can be beyond the MIR diffraction limit due to the visible-light-based PC microscopic detection of the photothermal optical-phase change. In addition, the wide-field detection allows for high-speed image acquisition ultimately limited by the frame rate of the image sensor. This can be beyond the reach of the state-of-the-art label-free chemical imaging methods such as coherent Raman imaging^17,18^ in which a point-scanning mechanism is typically used for image acquisition, limiting its imaging speed to ~30 fps. Furthermore, the sample’s photodamage associated with linear and nonlinear electronic transitions^19^ could be significantly reduced with our technique due to the low photon energy of MIR light as well as the low optical fluence of the visible light used for wide-field PC microscopic observation.

### High-speed molecular-vibrational imaging of microbeads

**Figure 2:**
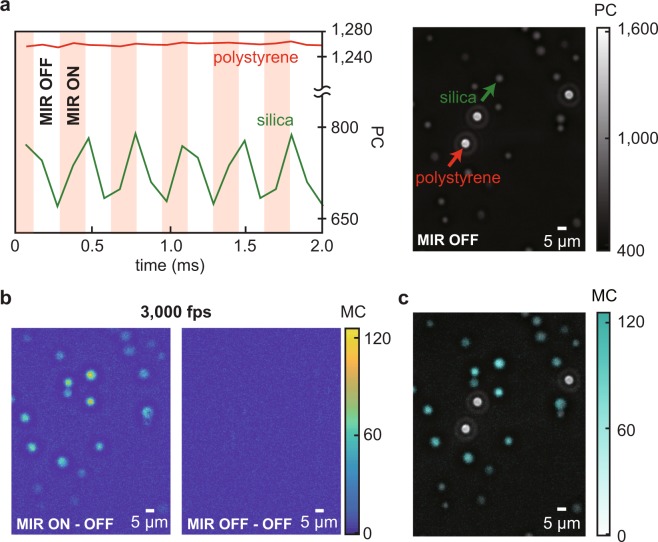
High-speed wide-field bond-specific imaging with the MC-PC microscope. (**a**) Site-specific time-dependent PC signals. The shown signals are the spatial-average of 6 × 6 pixels (3 μm × 3 μm) at the center of the silica (green curve and arrow) and the polystyrene (red curve and arrow) microbeads. The right panel shows a standard PC microscope image. (**b**) MC image obtained at 3,000 fps, corresponding to one MIR excitation cycle. The SNR is 13.2. Note that there exists a systematic spatial variation of the MC due to the spatial intensity profile of the excitation MIR beam spot at the sample plane. Left: MIR ON – OFF image. Right: MIR OFF – OFF image. **c**, MC-PC image synthesized by overlaying the MC (obtained in **b**) on top of the conventional PC image (shown in **a**). The MC selectively highlights the locations of the resonant porous silica microbeads out of the mixture sample, providing additional but powerful information on top of the conventional PC image.

To demonstrate the bond-specific molecular contrast of our MC-PC microscope, we measure a mixture of polystyrene and porous silica microbeads with a diameter of ~5 μm immersed in index-matching oil with the MIR beam configured to 1,045 cm^−1^ (resonant to Si-O-Si stretch of SiO_2_). Figure 2a shows the temporal PC intensities of the polystyrene and the porous silica microbead. The former stays constant while the latter shows periodic modulation at a frequency of 3,000 Hz which is the modulation frequency of the MIR beam. This clearly shows the modulated MIR beam induces the bond-specific PC intensity modulation. Shown in Fig. 2b is the MC image obtained in one MIR excitation cycle, visualizing the two-dimensional locations of the porous silica microbeads. The obtained MC images include spurious signal that originates from other mechanisms than the photothermal effect, such as Halo effect of the PC microscope, which can be filtered as detailed in the Supplementary Information. The wide-field image-acquisition rate is 3,000 fps for measuring ~200 × 200 of diffraction-limited pixels (~100 μm × 100 μm area) and could be further increased with a higher MIR modulation frequency and higher-power MIR light sources. Finally, Fig. 2d shows the MC-PC image synthesized by overlaying the obtained MC on top of the conventional PC image, where the porous silica microbeads are selectively highlighted.

### Characterization of MC-PC microscopy

**Figure 3:**
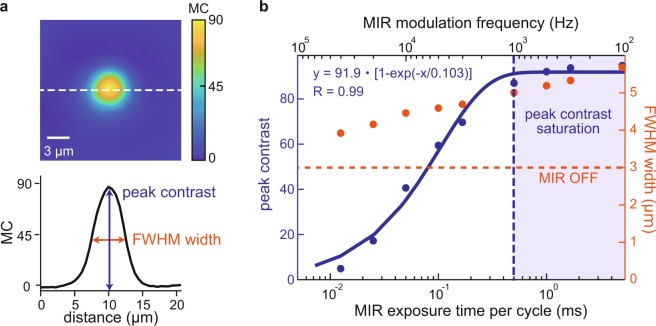
MIR modulation frequency vs MC, frame rate and spatial resolution. (**a**) MC image of a 5-μm porous silica microbead immersed in the index-matching oil used to characterize the system performance. The peak contrast and the FWHM width are used to analyze the system performance. (**b**) Performance characterization of the MC-PC microscope. Blue dots: peak contrast. Orange dots: FWHM width of MC images. Orange dashed line: FWHM width of the standard PC image at the complete MIR-OFF state. Blue dashed line: MIR frequency at which the peak-contrast saturates.

We characterize the performance of our MC-PC microscope in terms of the MC, frame rate and spatial resolution. Shown in Fig. 3 is how the MC and the full-width-at-half-maximum (FWHM) width of a 5 μm porous silica microbead in the index-matching oil (Fig. 3a) change with different MIR modulation frequencies (or MIR exposure time per modulation cycle). Note that the modulation frequency translates to the maximum possible frame rate to obtain the MC image. If the camera’s frame rate is kept the same, the maximum possible frame rate is in trade off with the SNR, e.g., when capturing at 100,000 fps, 1,000 Hz MIR modulation frequency translates to 1,000 fps at maximum for the MC-image acquisition by averaging 100 frames, whereas 100 Hz MIR modulation frequency translates to 100 fps at maximum for the MC-image acquisition by averaging 1,000 frames.

According to the graph shown in Fig. 3b, the MC increases with a longer MIR exposure and saturates above 0.5 ms, due to the existence of the heat diffusion time τ ∝ 1 ∕ α (α: thermal diffusivity of the heat absorber)^20,21^. When the MIR exposure is longer than τ, the supplied heat accumulates to balance with the heat diffusion, building a strong saturated MC. Such phenomenon can be modeled by a rate equation, and our measurement data indeed fits well to an exponential function (τ ~ 100 μs). The heat diffusion time could vary depending on the size of interest. Many liquid, polymer and glass materials have α ~ 10^−7^ [m^2^/s] (e.g., paraffin: ~0.8 × 10^−7^, ethanol: ~0.85 × 10^−7^, water: ~1.4 × 10^−7^, polycarbonate: ~1.5 × 10^−7^, glass: ~5.6 × 10^−7^)^22–24^, suggesting that similar results could be replicated in other experimental conditions. On the other hand, the MC FWHM width becomes larger than that of the original PC image (~3 μm) and increases with a longer MIR exposure. The degradation of the spatial resolution is due to heat diffusion towards the surrounding medium in the MIR ON state and, in the case of high MIR modulation frequencies, insufficient cooling in the MIR OFF state. To mitigate these two effects and achieve diffraction-limited spatial resolution of the visible light, pulsed MIR excitation and visible probe illumination are needed as well as a sufficiently long time-interval between two pump-probe measurements for the cooling which is ~1 ms in this case.

### Molecular-vibrational spectroscopic imaging of HeLa cells

**Figure 4:**
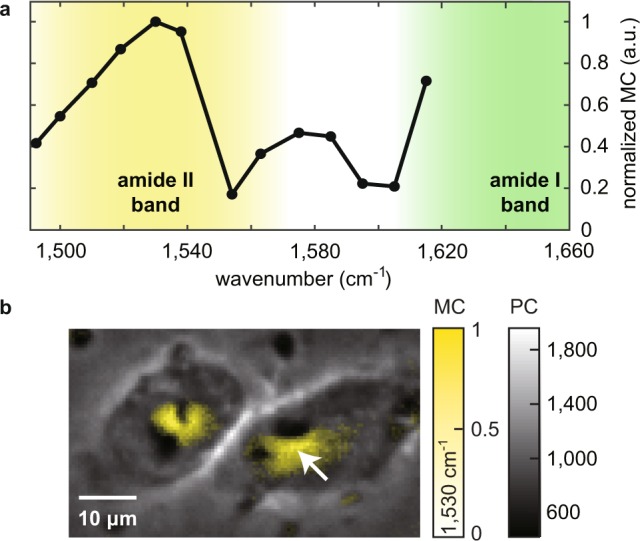
Molecular-vibrational spectroscopic imaging of HeLa cells measured with the MC-PC microscope. (**a**) MC spectrum of the HeLa cell obtained at the spatial point indicated by the white arrow in (**b)**, showing the characteristic curve of a HeLa cell representing the amide I and II bands of peptide bonds of proteins. The yellow and green shaded areas represent the amide II and I band, respectively. (**b**) MC image (yellow scale) of the HeLa cells measured under the vibrational excitation by the MIR beam lasing at 1,530 cm^−1^ overlaid on top of the standard PC image (gray scale), highlighting the intracellular protein distribution. The MC image is obtained at 10 fps (i.e., 100 ms acquisition time).

Now we show the ability of our MC-PC microscope to perform molecular-vibrational spectroscopic imaging by obtaining the MC images of HeLa cells at various MIR wavenumbers ranging from 1,492.5 to 1,615 cm^−1^. We note the refractive-index change arising from the MIR absorption of water, compared to that of e.g., proteins, should be small due to its high heat capacity and weak temperature dependence of the refractive index^13^; however, in this proof-of-concept demonstration, to achieve efficient delivery of the MIR light to the cells, we replace the surrounding medium with deuterated water. Figure 4a shows the MC spectrum measured at the spatial point in the cell indicated by the white arrow in Fig. 4b, where the MC values are normalized by the corresponding MIR power (see Supplementary Information for the linearity of MC with MIR power). The curve shows a good agreement with a characteristic spectrum of a HeLa cell^25^ which has the broad absorption bands of the peptide bonds of various proteins (i.e., amide II and I bands) ranging between ~1,500–1,580 and ~1,580–1,700 cm^−1^ and peaked at ~1,530 and ~1,650 cm^−1^, respectively.

Our MC-PC microscope offers the spatial resolution based on the visible light (~1 μm) which is an order of magnitude higher than that of MIR absorption imaging (~10 μm) and allows us to visualize the intracellular distribution of molecular species. Shown in Fig. 4b, for example, is the MC image of 1,530 cm^−1^ MIR wavenumber overlaid on top of the standard PC image, highlighting the intracellular protein distribution of the HeLa cells. In terms of the sensitivity and acquisition-speed, each MC image is obtained at 10 fps (100 ms acquisition time) with the highest SNR of 12.6. Since our microscope is based on a standard PC microscope, the fluence of the visible probe light, which is known to induce photodamage to biological samples^19,26^, is orders of magnitude lower (~630 pJ/μm^2^) than those of other molecular imaging techniques such as fluorescence and Raman imaging. The frame rate can be further increased using a higher-power MIR light source, as well as with a higher camera frame rate and/or at the expense of the SNR (see Supplementary Information for more detail).

It is of importance that the dual-modal MC-PC imaging capability is a unique feature of our microscope which allows us to determine the molecular distributions within the global morphology of the sample from a single dataset. This is not readily achieved by conventional imaging modalities because optical-phase-sensitive (i.e., morphological)^4^ and molecular-sensitive imaging^17,18,27^ cannot be performed without the loss of spatiotemporal consistency. In Fig. 4b, for example, the protein concentration (yellow) can be observed to be higher at the center and around the nucleus of the cells, which could represent the existence of subcellular organelles such as endoplasmic reticulum and Golgi apparatus, which are known to localize near the cell’s nucleus in other Raman microscopy works^28,29^. Such cross-correlative analysis would be more interesting with the addition of other distinct biological contrasts such as MCs resonant to lipids and deoxyribonucleic acids (DNAs), which is readily achievable by implementing MIR light sources of other wavenumber ranges (e.g., ~1,740 cm^−1^ for >C=O ester stretch representing lipids, ~1,080 or 1,240 cm^−1^ for O-P=O stretch representing DNAs, etc.)^30^. Overall, this biological demonstration suggests the potential ability of our MC-PC microscope to perform high-speed, high-resolution and low-power molecular-vibrational spectroscopic imaging.

## Discussion

The novelty of our MC-PC microscopy in the context of photothermal microscopy is the wide-field MIR excitation of the photothermal phenomenon and its visible-light-based detection, which could potentially lead to higher-speed image acquisition. In conventional MIR photothermal microscopy techniques^13–15^, the thermal lensing deflection of the visible probe beam due to MIR photothermal effect is probed in a point-by-point manner. To avoid the accumulation of heat (which leads to degraded spatial resolution as discussed in “Characterization of MC-PC microscopy” section), one needs to wait for the supplied heat to diffuse away from the current excitation point before probing the next neighboring spatial point. This means that the shortest image-acquisition time possible is the product of the thermal decay time and the pixel number. The same issue regarding the heat diffusion also needs to be considered in the case of our MC-PC microscopy; however, due to the wide-field excitation and detection, entire excited area experiences the synchronized thermal decay, meaning that the shortest image-acquisition time possible is identical to the thermal decay time.

There are some limitations to the sample that can be observed with the MC-PC microscopy. For example, sufficient penetration of the visible and MIR light through the sample (which, in the case of biological samples, would mainly be water) needs to be guaranteed such that both beams reach the molecules of interest, which could be realized with, e.g., living cells^13^ and drug tablets^14^. Even when the surrounding medium absorbs part of the MIR light and shows the undesired photothermal signal, multi- or broadband spectroscopic data could be used to decouple such effect and enhance the molecular specificity.

Several technical improvements could be considered to make our system more robust and convenient. It is desirable to homogenize the spatial profile of the MIR beam at the sample plane so that the systematic distribution of the MC is physically removed, which could be realized using, e.g., diffractive-optical elements^31^. Another possible improvement is to collinearly introduce the MIR excitation and the visible probe beams using, e.g., a reflective condenser lens, for easier and possibly automated alignment of the MIR beam. Our MC unit could be packed with a reflective condenser lens and a probe light source to serve as a compact add-on module to existing optical microscope platforms. The sensitivity of our MC-PC microscope could also be further enhanced. Standard benchtop optical microscopes are typically shot-noise limited due to the active illumination resulting in a large number of detectable photons, suggesting that the camera’s full-well capacity and frame rate become the ultimate limitations. Technological advancement in the camera industry would then lead to an enhanced sensitivity with the MC-PC microscope. Other imaging modalities could also be used, such as dark-field, differential-interference-contrast and other quantitative phase imaging techniques^4^, some of which can also be operated on a standard benchtop optical microscope.

## Methods

### Principle of MC-PC microscopy

We used the following instruments to construct our MC-PC microscope: (1) PC microscope: LUCPLFLN 40XPH and IX73 (Olympus), (2) MIR QCL light sources: QD9500CM1 (Thorlabs) configured to 1,045 cm^−1^ for experiments with microbead samples and DO418, Hedgehog (Daylight Solutions) for spectroscopic experiments with HeLa cells (spectral coverage: 1,450–1,640 cm^−1^, spectral resolution: ~1 cm^−1^, spectral tuning speed: >1,000 cm^−1^/s), (3) camera: MEMRECAM HX-7s (Nac) and (4) visible LED light source: SOLIS-623C (Thorlabs). The MIR beam is weakly focused onto the sample placed at the objective focus using a CaF_2_ lens. We avoid tight focusing of the MIR beam in order to illuminate and excite the entire region of the sample (~100 μm × 100 μm). The sample is sandwiched between two 500-μm-thick CaF_2_ substrates in order to avoid absorption of MIR light by the substrate medium. The amplitude of the MIR light is modulated either electrically (QD9500CM1) or using an optical chopper (DO418) at a desired rate.

### Experimental conditions

We used the following materials for our experiments: (1) 4.8-μm polystyrene microbeads: 17135-5, Polybead Microspheres (Polysciences, Inc.), (2) 5-μm porous silica microbeads: 43-00-503, Sicastar (micromod Partikeltechnologies GmbH), and (3) index-matching oil: refractive index 1.50 at 587.56 nm (SHIMAZU). The visible LED power is ~50 μW at the sample plane.

### MC image synthesis procedure

The MC image is obtained from the time-sequence of MIR-modulated PC images by calculating the difference of two averaged images; the ones calculated from the time-frames showing the lower-half (i.e., MIR-OFF image) and the higher-half (i.e., MIR-ON image) magnitudes of the photothermal PC modulation at the MIR absorbers. The spurious negative signal in the MC image (which does not directly originate from the photothermal effect) is eliminated using the negative-contrast filter described in Fig. S2.

### High-speed imaging on the silica-polystyrene microbeads mixture

The MIR modulation frequency is 3,000 Hz and the camera frame rate is 10,000 fps. To derive the SNR, the noise level is determined by the standard deviation of the empty region (20 × 20 pixels, 12 μm × 12 μm) of the MC image before the negative-contrast filtering, while the signal level is determined by the averaged MC at the center of the microbeads (5 × 5 pixels, 3 μm × 3 μm) where the strongest MC is observed. The MIR power is ~10 mW.

### Characterization of MC-PC microscopy

For each MIR modulation frequency, the microbead’s MC image is retrieved from a continuous series of 5,000 PC images captured at the frame rate of 100,000 fps (measurement time 0.05 s). This gives the same noise floor for the MC image of each MIR modulation frequency. To retrieve the peak contrast and the FWHM width, each of the obtained MC images is interpolated by a factor of 4 in each spatial dimension, to create its smooth cross-sectional curve.

### Spectroscopic imaging of HeLa cells

The HeLa cells were fixed with 4% paraformaldehyde at room temperature (5 min.) and immersed in deuterium oxide. The MIR modulation frequency is 250 Hz and the camera frame rate is 10,000 fps. A continuous series of 1,000 images is used to calculate each MC image, corresponding to 25 cycles of MIR modulation and the acquisition time of 100 ms. The SNR is calculated based on the MC image at 1,530 cm^−1^. The noise level is determined by the standard deviation of the empty region (~25 × 25 pixels, 14 μm × 14 μm) in the MC image before the negative-contrast filtering, while the signal level is determined by the averaged MC at the center of the HeLa cell (~5 × 5 pixels, 3 μm × 3 μm) where the strongest MC is observed. The spectral resolution is determined by the wavelength-tunability of the light source, which is ~1 cm^−1^ in our case although we use 10 cm^−1^ increment in this experiment. The MIR power is dependent on the wavenumber and varies between ~16–40 mW.

## Supplementary information

Supplementary Information

## Data Availability

The datasets generated and analyzed in the current study is available upon request to the authors.
