# Supplementary material for: Molecular contrast on phase-contrast microscope
Source: Sci Rep. 2019 Jul 18;9:9957. doi: 10.1038/s41598-019-46383-6 (PMC6637114; doi:10.1038/s41598-019-46383-6)
Supplement: Supplementary file 1 — Supplementary Information [file 41598_2019_46383_MOESM1_ESM.docx]

**Supplementary Information:**

**Molecular contrast on phase-contrast microscope**

**Keiichiro Toda,^1,†^ Miu Tamamitsu,^1,†^ Yu Nagashima,^2^ Ryoichi Horisaki,^3,4^ and Takuro Ideguchi^4,5,*^**

^1^Department of Physics, The University of Tokyo, Tokyo 113-0033, Japan

^2^Department of Neurology, The University of Tokyo, Tokyo 113-0033, Japan

^3^Graduate School of Information Science and Technology, Osaka University, Osaka 565-0871, Japan

^4^PRESTO, Japan Science and Technology Agency, Saitama 332-0012, Japan

^5^Institute for Photon Science and Technology, The University of Tokyo, Tokyo 113-0033, Japan

^*^Corresponding author: ideguchi@gono.phys.s.u-tokyo.ac.jp

^†^These authors contributed equally to the work.

**Linearity of MC vs MIR excitation power**

To verify the response of the MC with respect to the MIR excitation power, we obtain the MC images of HeLa cells immersed in deuterium oxide at 1,554 cm^-1^ with various MIR powers ranging between ~ 5 – 27 mW over the area of ~ 100 µm × 100 µm, and plot the obtained MC. The MIR modulation frequency is 250 Hz and the camera frame rate is 10,000 fps. A continuous series of 10,000 images is used to calculate each MC image, corresponding to 250 cycles of MIR modulation and the acquisition time of 1 s. The MC in each image is calculated by averaging 2 × 2 pixels (~ 1 µm × 1 µm) region of the center part of the cell. The result shown in **Fig. S1** verifies the linear relation.


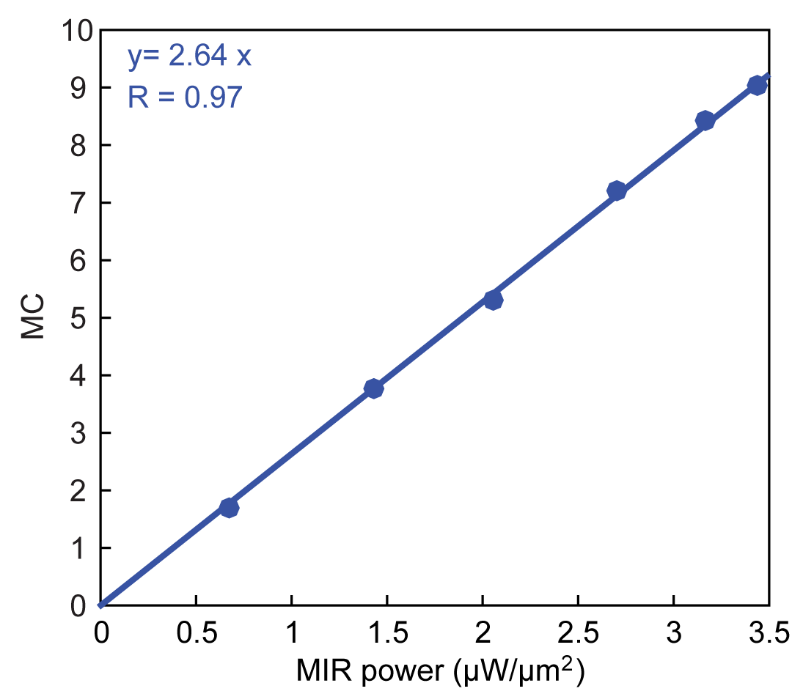


**Figure S1. Linearity of MC vs MIR excitation power with the MC-PC microscope.**

**Filtering spurious negative signal in MC image**

In our MC-PC measurement, a spurious negative signal is observed in the MC image. This negative MC is not the direct consequence of the photothermal effect but other phenomena such as Halo effect accompanied by the PC measurement. **Fig. S2a** and **b** show the PC and MC images of the HeLa cells discussed in **Fig. 4** in the main text, respectively. As seen in the MC image, we can clearly see the negative-MC region around the positive-MC region. To visualize the direct photothermal effect (the positive MC) only, we filter off the spurious negative contrast with a binary mask shown in **Fig.** **S2c**. The filtered MC is shown in **Fig. 4b** in the main text.


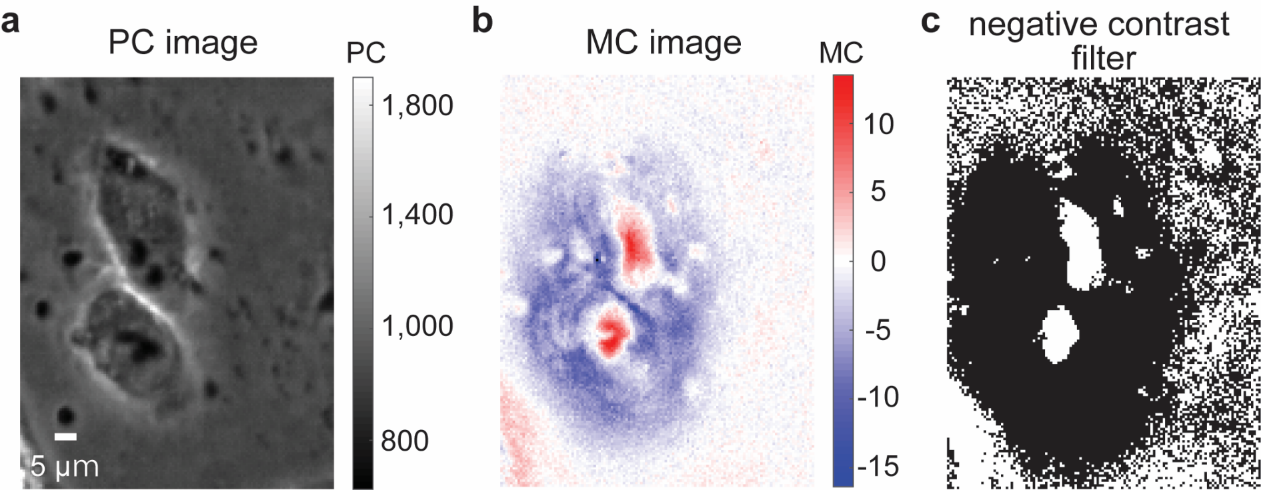


**Figure S2. Filtering spurious negative signal in MC image. a**, Standard PC microscopic image of the HeLa cells. **b**, MC image. **c**, Negative-contrast filter (binary mask).

**MC frame rate and SNR**

The limitation of the MC frame rate, $F_{\mathrm{MC}}$, is given by the camera frame rate, $F_{\mathrm{camera}}$, and the MIR modulation frequency, $F_{\mathrm{MIR}}$, by the following equation:

F_MC_ ≤ F_MIR_ ≤ F_camera_ ∕ 2 (S1)

The first inequality represents that at least one cycle of MIR modulation is necessary to obtain the MC image, whereas the second the Nyquist-Shannon theorem for sampling the MIR modulation with the camera. Therefore, the ultimate limitation of the MC frame rate is determined by the camera frame rate. The higher MC frame rate, however, generally results in a lower SNR. In terms of the noise, the noise level increases with an increased MC frame rate, due to reduction in the number of averaged frames per MIR excitation cycle. In terms of the signal, the MC shows exponential decrease with a higher MIR modulation frequency below the saturation level as shown in **Fig. 3** in the main text. To increase the SNR, the following modifications can be made. For a fixed MIR modulation frequency, a higher MC can be obtained with a higher MIR power (realized by e.g., having a higher output from the MIR light source, focusing the MIR beam tightly, etc.) as the photothermal optical-phase change is linear to the input MIR power (see **Fig. S1**). The higher SNR can also be obtained with image sensors with a higher full-well capacity in the condition dominated by the optical shot noise.

**Detailed analysis on the spectroscopic images of HeLa cells**

With a more detailed analysis on the obtained spectroscopic images, there is a chance to reveal the existence of other molecular species in HeLa cells than proteins within the measured MIR wavenumber range. **Fig. S3a** shows the normalized MC spectra measured at three different spatial points in the cells indicated by the red, green and blue arrows in **Fig. S3b**. The blue curve represents a characteristic spectrum of a HeLa cell representing the amide II and amide I bands. Indeed, as shown in **Fig. S3c**, the MC images at 1,530 and 1,615 cm^-1^ show nearly identical profiles, indicating these MCs originate in the same type of molecular species (i.e., proteins). On the other hand, the red and green curves show peculiar peaks at 1,500 and 1,554 cm^-1^, respectively, and the MC images at 1,500 and 1,554 cm^-1^ show characteristic distributions different from that of the proteins, respectively. Shown in **Fig. S3d** is an overlay of the three MC images at 1,500, 1,530 and 1,554 cm^-1^ on top of the standard PC image, highlighting the different MC spatial distributions. Here, the protein concentration (blue) can be observed to be higher at the center and around the nucleus of the cells while distributions of the other molecular species seem to follow some cellular structures recognized in the PC image (see, e.g., the green and red contrasts found on the protruding structures recognized at the bottom right corner of the top cell and the left side of the bottom cell, respectively). Although we have not assigned the origins of the spectral features of 1,500 and 1,554 cm^-1^ due to the lack of literature, we could have a chance to find new information.


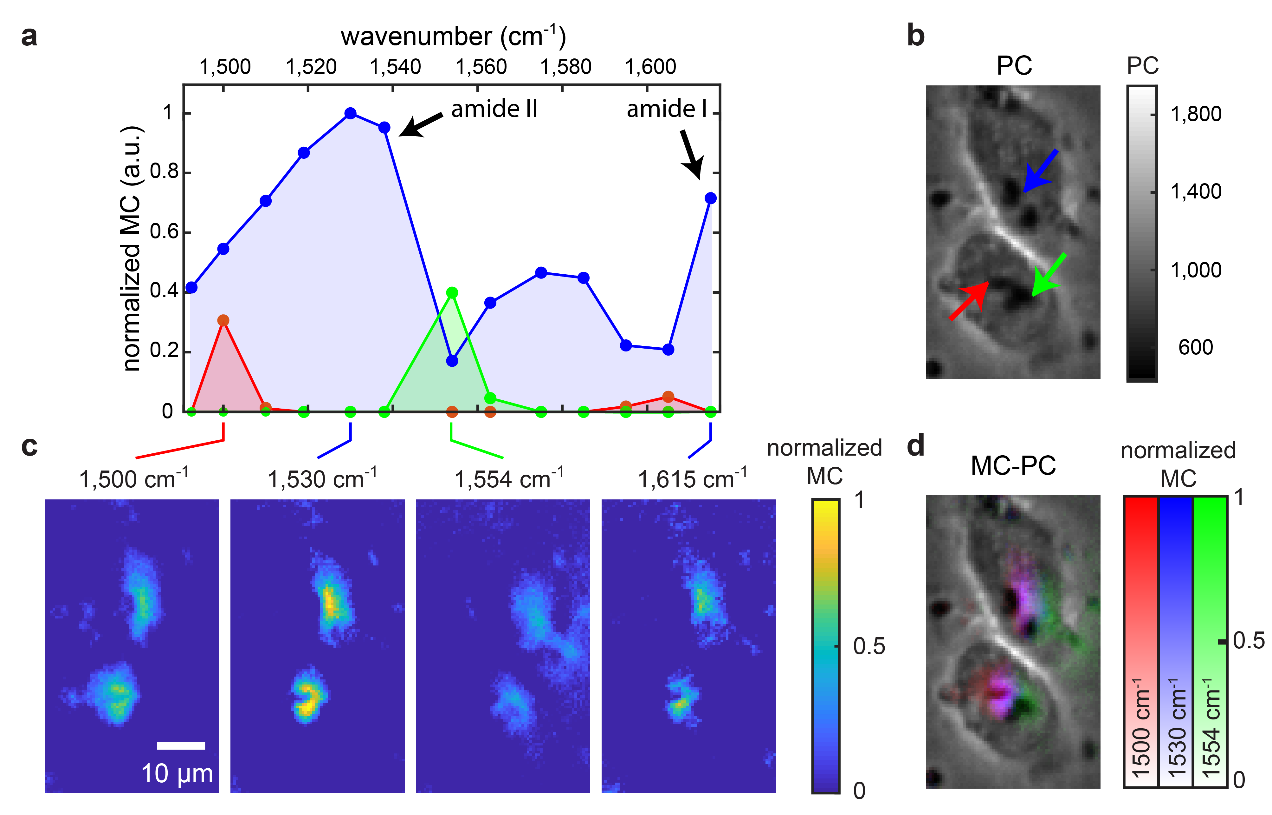


**Figure S3. Detailed analysis on the molecular-vibrational spectroscopic images of HeLa cells. a**, MC spectra of HeLa cells obtained at the spatial points indicated by the arrows in **b**. The colors of the data points correspond to those of the arrows in **b**. **b**, PC image of the HeLa cells obtained at the MIR-OFF state, similar to a standard PC microscopic image. **c**, MC images of the HeLa cells measured under the vibrational excitation by the MIR beam lasing at 1,500, 1,530, 1,554 or 1,615 cm^-1^. Each MC image is obtained at 10 fps (i.e., 100 ms acquisition time). **d**, MC-PC image of the HeLa cells comprised of the MC images at 1,500, 1,530 and 1,554 cm^-1^.
